# Free Electron Density Gradients Enhanced Biosensor for Ultrasensitive and Accurate Affinity Assessment of the Immunotherapy Drugs

**DOI:** 10.1002/advs.202404559

**Published:** 2024-10-23

**Authors:** Youqian Chen, Hongli Fan, Rui Li, Huazhi Zhang, Rui Zhou, Gang L. Liu, Chunmeng Sun, Liping Huang

**Affiliations:** ^1^ College of Life Science and Technology Huazhong University of Science and Technology 1037 Luo Yu Road Wuhan 430074 P. R. China; ^2^ Biosensor R&D Department Liangzhun (Wuhan) Life Technology Co. Ltd Wuhan 430070 P. R. China; ^3^ Department of Pharmaceutics School of Pharmacy China Pharmaceutical University 639 Longmian Avenue Nanjing 211198 P. R. China; ^4^ NMPA Key Laboratory for Research and Evaluation of Pharmaceutical Preparations and Excipients China Pharmaceutical University 24 Tong Jia Xiang Nanjing 210009 P. R. China; ^5^ School of Food Science and Pharmaceutical Engineering Nanjing Normal University Nanjing 210023 P. R. China

**Keywords:** affinity, free electron density, immunotherapy drugs, label‐free detection, MetaSPR, molecular interactions

## Abstract

Accurate affinity assessments play an important role in drug discovery, screening, and efficacy evaluation. Label‐free affinity biosensors are recognized as a dependable and standard technology for addressing this challenge. This study constructs a free electron density gradient‐enhanced meta‐surface plasmon resonance (FED‐MSPR) biosensor through a finite‐difference time‐domain simulation model, the biosensor demonstrates superior detection performance in accurately determining affinity and kinetic rate constants. By controlling the dielectric properties of the metal on the surface of the nanocup arrays, the plasmon resonance effects are easily tuned without changing the nanostructure design. Compared with the single‐layer gold chip, the triple‐layer FED‐MSPR chip demonstrated a four‐fold improvement in resolution at the optimal resonance peak. Additionally, the sensitivity and figure of merit (FOM) of the multi‐layer chip increased by 3.5 and 7.99 times, respectively. Following modification with high‐ and low‐staggered carboxylation, the noise‐signal ratio and baseline stability of the real‐time kinetic curves based on these chips are significantly enhanced. The developed carboxylation FED‐MSPR platform is successfully used to perform affinity assays for Adalimumab and TNF‐α protein, resulting in favorable dynamic curves. These findings validate the proposed FED‐MSPR biosensor platform as cost‐effective, rapid, sensitive, and label‐free, facilitating real‐time quality control in drug development.

## Graphical abstract

This study proposes a free electron density gradient‐enhanced meta‐surface plasmon resonance (FED‐MSPR) biosensor with extraordinary detection performance in accurately determining affinity and kinetic rate constants. The developed carboxylation FED‐MSPR biosensor is successfully used to perform affinity assays for adalimumab and TNF‐α protein, validate the proposed FED‐MSPR biosensor platform as cost‐effective, rapid, sensitive, and label‐free, facilitating real‐time quality control in drug development.

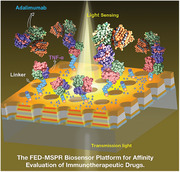

## 1 Introduction

The utilization of antibody drugs in treating autoimmune diseases is well‐documented,^[^ ^1^ ^]^ which Adalimumab targeting tumor necrosis factor‐alpha (TNF‐α) at the forefront of managing conditions such as ankylosing spondylitis,^[^ ^2^ ^]^ rheumatoid arthritis,^[^ ^3^ ^]^ and psoriasis.^[^ ^4^ ^]^ These drugs selectively target cytokines closely related to the development of the diseases, potentially circumventing the common adverse effects of traditional treatment.^[^ ^5^ ^]^ Immunotherapy's rise^[^ ^6^ ^]^ underscores the need for sensitive and specific methods to assess drug affinity and interaction kinetics, which are paramount for ensuring therapeutic efficacy and safety.^[^ ^7^ ^]^ Therefore, affinity assessment becomes pivotal in determining immune drug quality.

Surface plasmon resonance (SPR) technology is a versatile technique widely used in designing optical sensing platforms^[^ ^8^ ^]^ and extensively employed in molecular interaction research, including antigen–antibody, drug‐target,^[^ ^9^ ^]^ protein–nucleic acid,^[^ ^10^ ^]^ protein–DNA,^[^ ^11^ ^]^ protein–protein,^[^ ^12^ ^]^ and protein–lipid^[^ ^13^ ^]^ interactions, by monitoring the refractive index change on the sensor surface caused by the surface load changes.^[^ ^14^ ^]^ SPR technology holds paramount significance in immunological research, providing real‐time insights into the affinity and kinetics of biomolecular interactions.^[^ ^15^ ^]^ Consequently, they have emerged as indispensable analytical tools for real‐time detection across various domains.^[^ ^10^, ^16^ ^]^ Meta‐surface plasmon resonance (MetaSPR) technology is an innovative technique compared to SPR, leveraging metamaterials to induce resonance in free electrons within metal nanostructures. MetaSPR technology operates on the principle that incident light on optical metal nanostructures induces resonance of free electrons in the metal. This electronic resonance leads to secondary light emission, resulting in the formation of distinct transmission spectra.^[^ ^17^ ^]^ Molecular interactions on the chip surface can induce alterations in the plasmon resonance transmission spectrum, causing a significant change in transmitted light intensity at two specific wavelengths.^[^ ^15b^ ^]^ Therefore, the detection of biomolecular interactions involves monitoring changes in the transmitted light intensity at two wavelengths, contrasting with traditional SPR techniques reliant on alterations in the resonance angle of the reflected light.^[^ ^18^ ^]^ The MetaSPR technology offers distinctive benefits in biomolecular detection^[^ ^19^ ^]^ mainly encompassing the multifaceted design of nanoarray structures, a diverse palette of metamaterial choices, broad spectral tunability, significant amplification of localized electric fields, and simplified optical detection modalities, among others. However, in practical molecular interaction assays, the insensitivity of the sensor to minute refractive index (RI) changes, coupled with the potential for non‐specific effects, increases the difficulty of directly detecting biomolecules using a MetaSPR chip. Therefore, the detection sensitivity and stability of MetaSPR still need to be further improved to meet the detection requirements for molecules with either very high or very low affinities, as well as for molecules with very small molecular weights.^[^ ^20^ ^]^ Numerous studies have been dedicated to enhancing the detection sensitivity of MetaSPR through the optimization of nanostructures,^[^ ^21^ ^]^ surface modifications,^[^ ^22^ ^]^ and detection systems.^[^ ^23^ ^]^ This research, however, takes a novel approach by meticulously adjusting the dielectric characteristics of the metal on the nanocup arrays' surface to enhance the detection performance of MetaSPR.

It is widely acknowledged that metals and metal oxides possess the ability to enhance the detection sensitivity of biosensors. The surfaces of metals and metal oxides are susceptible to the adsorption of unstable organic materials, potentially altering the free energy of the interface between the metals or metal oxides and their surroundings. Therefore, the choice of metal layers plays a significant role in achieving higher sensor sensitivity, with gold and silver being primary contenders. Gold, prized for its exceptional stability,^[^ ^24^ ^]^ is a cornerstone material in the construction of SPR biosensors,^[^ ^25^ ^]^ with the majority employing thin gold films.^[^ ^26^ ^]^ Despite this, the sensitivity of a single gold film is insufficient for detecting biomarkers at low concentrations. While silver offers superior electric field enhancement and deeper penetration, increasing sensitivity^[^ ^27^ ^]^ However, the susceptibility of Ag to oxidation and sulfurization limits some of its SPR properties. Given the distinct dielectric constants of gold (Au) and silver (Ag), their strategic combination can result in a synergistic enhancement of the plasmonic effects. The use of an Au‐coated Ag metal layer^[^ ^20b^ ^]^ as the sensing layer is a testament to this, where the gold film acts as a protective layer for the silver coating. This approach not only enhances the sensitivity by leveraging the high electron density of silver but also introduces the stability and biocompatibility of gold, preventing oxidation of the Ag coating. By integrating these materials into the nanocup array structure, as we previously developed,^[^ ^21^ ^]^ the specific surface area of the chip is effectively increased, boosting the load capacity and improving the response signal. The unique interplay between the plasmonic properties of these two metals in the nanocup array structure offers an opportunity to optimize the sensor performance of the MetaSPR sensor. However, mapping the number of metal layers based on the nanocup array structure has not been studied. Therefore, this study aims to explore the optimal metal‐layer structure of the MetaSPR sensor chip to achieve higher detection sensitivity and improved sensing performance, for label‐free affinity assessment for immunotherapeutic drugs.

**Figure 1:**
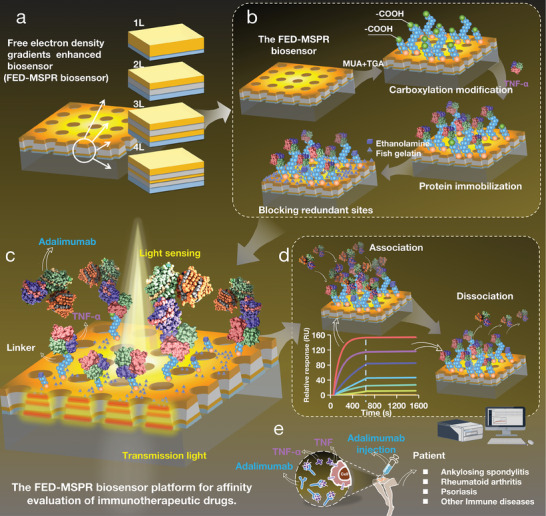
Schematic diagram of workflow and principle of immunotherapeutic drug affinity assessment using the FED‐MSPR biosensors. a) Design scheme of MetaSPR chip with nanocup array structure of single‐layer (1L) metal structure chip, double‐layer (2L), triple‐layer (3L), and quadruple‐layer (4L) chips. b) Schematic diagram of the carboxylation process on the surface of the FED‐MSPR chip. c) Schematic diagram of MetaSPR technology platform for evaluating the affinity of immunotherapy drugs. d) Process and principles of immunotherapeutic drug affinity assessment. e) Significance of affinity assessment of immunotherapeutic drugs.

In this study, we utilized finite‐difference time‐domain (FDTD) simulations to optimize the design of MetaSPR chip sensors with varying free electron density metal gradients. We fabricated MetaSPR chips with single‐layer (Ti‐Au), double‐layer (Ti‐Ag‐Au), triple‐layer (Ti‐Au‐Ag‐Au), and quadruple‐layer (Ti‐Ag‐Au‐Ag‐Au) configurations via evaporation from a PET film with a specific nanocup array structure. Through measurements of different sucrose solutions and affinity assessments between Protein A and human IgG, we demonstrated that the triple‐layer free electron density gradients (Ti‐Au‐Ag‐Au) chip demonstrated the highest sensitivity (**Figure** **1**a). Subsequently, we chemically modified the best‐performing free electron density gradients enhanced biosensor (FED‐MSPR biosensor) to enhance its surface functionality (Figure 1b), followed by an affinity assessment of the immunotherapeutic drug with its target protein (Figure 1c). The constructed affinity assessment platform utilizing the FED‐MSPR biosensor enables the evaluation of the binding activity of the TNF‐α protein with the Adalimumab drug in vitro (Figure 1d). This FED‐MSPR platform can be employed to evaluate the binding capacity and affinity, as well as the targeting specificity, of antibody therapeutics to their targets (Figure 1e). Our verification experiments confirm that the detection performance of the FED‐MSPR biosensor satisfies the practical application value in the affinity assessment of immunotherapeutic drugs. Furthermore, it underscores the significant potential of this technology in the development of monoclonal antibody drugs, promising a transformative approach to biopharmaceutical advancement.

## 2 Results and Discussion

### 2.1 Simulation of the Enhancement Effect of Free Electron Density Gradient

The frequency and intensity of surface plasmon absorption are influenced by the dimensions and morphology of the metal layer as well as the surrounding environment.^[^ ^28^ ^]^ Consequently, a comprehensive evaluation of the impact of metal structure on light absorption is essential when examining the sensing properties of MetaSPR chips. In this research, we simulated various metal combinations with different electron densities, using the adjustable properties of plasmon resonance peaks to analyze the performance of biosensors enhanced by electron density gradients.

Our simulations, informed by a conceptual model, clarify the role of free electron density gradients in enhancing light absorption and the sensing capabilities of MetaSPR chips. The interaction between layers in a multilayer system is hypothesized to lead to a constructive enhancement of the electric field at the nanocup's junctions, which is pivotal for plasmon resonance. This enhancement is theorized to depend on layer thickness, metal type, and the gradient of free electron density across layers.

**Figure 2:**
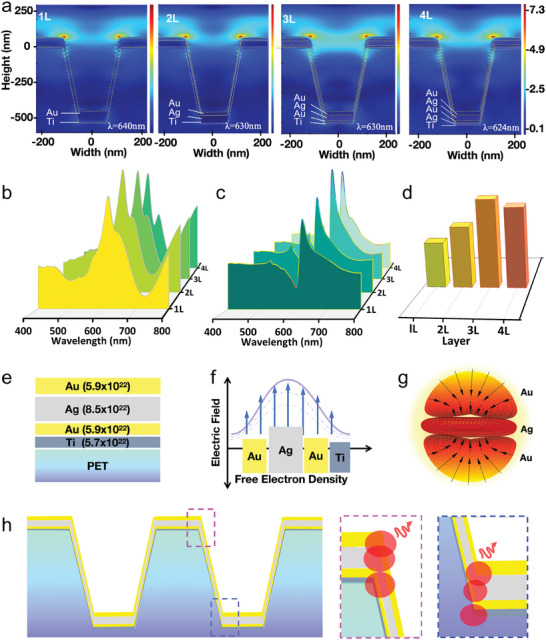
FDTD simulation and analysis of the resonance effect in FED‐MSPR sensors with varying electron densities. a) Legend displays 3D‐FDTD simulated electric fields for single to quadruple‐layer chips with varying free electron density gradients. b) Comparing the simulated absorption spectra of four chips, the triple‐layer chip has the highest simulated resonance peak. c) After water zeroing, the triple‐layer chip has the highest absorbance response, indicating the greatest sensitivity. d) The difference between the dual peaks simulating the change in resonance peaks, it can be observed that the triple‐layer chip shows the greatest response change to 5% sucrose. e) The nano‐structured configuration highlights the impact of different electron density gradients. f) Distribution of charge carrier density, illustrating the direction of energy flow between metal layers. g) Schematic showing the direction of the free electric field in the interface between gold and silver layers. h) Provides a schematic representation of the most active free electric field distribution region in sensors with different electron density combinations.

The 3D‐FDTD method is widely utilized for optical analysis of nanostructures in the design of nano‐optoelectronic devices. The electric field and absorbance of the four types of chips were simulated using 3D‐FDTD software. While no difference was observed in the electric field activity at the resonance peak (Figure S1, Supporting Information), a significant variation in the electric field at the valley (**Figure** **2**a) was observed. Compared with the single‐layer, double‐layer, and quadruple‐layer chips, the triple‐layer chip exhibited the most active electric field, with free electrons most active at the junction of the top and the wall of the nanocups. The simulated absorbance spectra further corroborate these findings, with the triple‐layer chip demonstrating the highest simulated resonance peak (Figure 2b). This peak is attributed to the optimized interaction between layers, amplifying the electric field and absorbance. These simulation results are well‐aligned with the experimental spectra, despite potential discrepancies due to factors such as device wettability.^[^ ^29^ ^]^ The differential spectra (Figure 2c) and the analysis of the peak and valley relative response (Figure 2d) underscore the superior sensitivity of the 3L system, which aligns with the experimental spectra and supports the physical model proposed.

Our simulation results demonstrate that the 3L chip, with its Au‐Ag‐Au nano‐structured configuration (Figure 2e), effectively forms a “low‐high‐low” free electron density gradient (Figure 2f). This gradient creates an electron density wave that concentrates the energy from the Au layer on the Ag layer, which has a high electron density, and the charge carrier density distribution is concentrated on the center Ag layer, resulting in a stronger free electric field (Figure 2g). In the 3L chip, the energy from the two Au layers converges on the central Ag layer, making the free electric field in the 3L chip more active than in the 2L and 1L chips. In contrast, the 4L chip, with an Ag‐Au‐Ag‐Au structure, has an additional layer of Ag with a high free electron density, which leads to energy diversion and a lower enhancement effect compared to the 3L chip. The interaction of nanoscale metals with light is characterized by surface‐bound charge density oscillations of free electrons resonating with the driving electromagnetic field.^[^ ^30^ ^]^ The curvature of charge density between these metal film layers can be understood in relation to the dielectric constant and charge density according to Poisson's equation:^[^ ^31^ ^]^

(1) $$p\;\left(x\right)=\varepsilon_{0}\;\varepsilon_{r}+\frac{d^{2}V_{S}}{dx^{2}}$$

where ε_0_ is the vacuum permittivity, ε_*r*_ is the relative permittivity and *x* is the lateral position.^[^ ^32^ ^]^

Equation (1) indicates that the frequency of MetaSPR is predominantly determined by the free electron density of the materials, which is regulated by its high‐frequency dielectric constant. In our Au‐Ag‐Au, silver exhibits a higher free electron density of 8.5 × 10^22^ cm^−3^ compared to gold's 5.9 × 10^22^ cm^−3^, resulting in a corresponding plasmon resonance effect in the visible spectrum. Titanium was selected for its favorable characteristics of low extinction coefficient and strong adhesion.^[^ ^33^ ^]^ Silver was selected for its favorable plasmon resonance and cost‐effectiveness,^[^ ^34^ ^]^ while gold was chosen for its visible plasmon resonance and superior stability.^[^ ^35^ ^]^ Consequently, Ti‐Au‐Ag‐Au sensors with varying electron density gradients exhibit optimal performance. The principle of this enhancement has been reported in the literature for similar A‐B‐A structures, such as in Au‐SiO_2_‐Au, Au‐Cds‐Au,^[^ ^29^ ^]^ Au‐TiO_2_‐Au,^[^ ^36^ ^]^ Ag‐SiO_2_‐Ag.^[^ ^37^ ^]^ In this work, the three‐layer chip structure allows for the convergence of electric charges from the Au layers on both sides toward the Ag layer in the center, thereby enhancing the chip's free electric field and improving sensing performance.

The inclusion of gold layers facilitates robust confinement of the electromagnetic field at the nanoscale. When the incident wavelength coincides with the plasmon resonance wavelength induced by the oscillation of unbound electrons under electric field (light) stimulation, it results in prominent surface plasmon absorption peaks and the amplification of the localized electromagnetic field (Figure 2h). The simulation results (Figure S2, Supporting Information) indicate the most intense distribution of the free electric field at the nanocup interlayer junctions, especially with the enhancement of electron densities in the Ti‐Au‐Ag‐Au layered chip. In the reported literature,^[^ ^22^, ^38^ ^]^ for chips based on Ti‐Ag‐Au, the most active regions of the free electric field are concentrated at the junctions of the nanocups. As the behaviors of the charge carriers play important roles in the photoelectric conversion process, researchers improve photoelectric performance by modulating the charge‐carrier behaviors.^[^ ^39^ ^]^ These are similar to the phenomenon we have discovered.

In summary, our simulations, supported by a conceptual model that the free electron density gradient, confirm that the 3L chip exhibits the highest sensitivity. This model provides a physical foundation for understanding the observed differences in sensitivity among various layer configurations and offers a framework for optimizing the design of MetaSPR chips for enhanced biosensing applications.

### 2.2 Durability Analysis and Long‐term Stability Discussion

We assessed chip durability using literature‐recommended methods,^[^ ^40^ ^]^ essential for SPR detection, which involves constant immersion in solutions like PBS. Following the stirring experiments with PBS, the surface roughness of the chips was compared via atomic force microscopy (AFM) (Figure S3, Supporting Information), with minimal change indicating higher stability. Preliminary inspections using optical microscopy and AFM showed all chips had high integrity, underpinning their durability. Initial surface roughness (Ra) values were 11.710, 5.438, 4.942, and 3.169, which shifted to 11.783, 6.235, 5.597, and 4.921 after PBS agitation (Figure S4, Supporting Information). It was observed that as the number of metal layers in the chip increased, the roughness of the chip decreased. After shaking with PBS, the surface roughness of the different multi‐layer free electron density chips did not change significantly, indicating that all the chips can remain highly stable.

Moreover, we further examined the stability of the chips under different conditions. Agitated in water solutions with pH 4.5, 6.0, 7.5, and 9.0 at 300 rpm for 48 h. The results (Figure S5a, Supporting Information) showed no degradation on the chip surface across all pH levels. Similarly, immersion in water at temperatures 4, 25, 37, and 42 °C for 48 h (Figure S5b, Supporting Information) revealed no flaking or damage, indicating that temperature and pH have negligible effects on chip stability. Research has shown that the multi‐layer free electron density chip inherits the sensitivity improvement observed in single‐layer chips but with significantly greater stability and uniformity, even in saline and acidic media,^[^ ^41^ ^]^ which is consistent with our experimental results.

### 2.3 Sensing Performance Analysis

**Figure 3:**
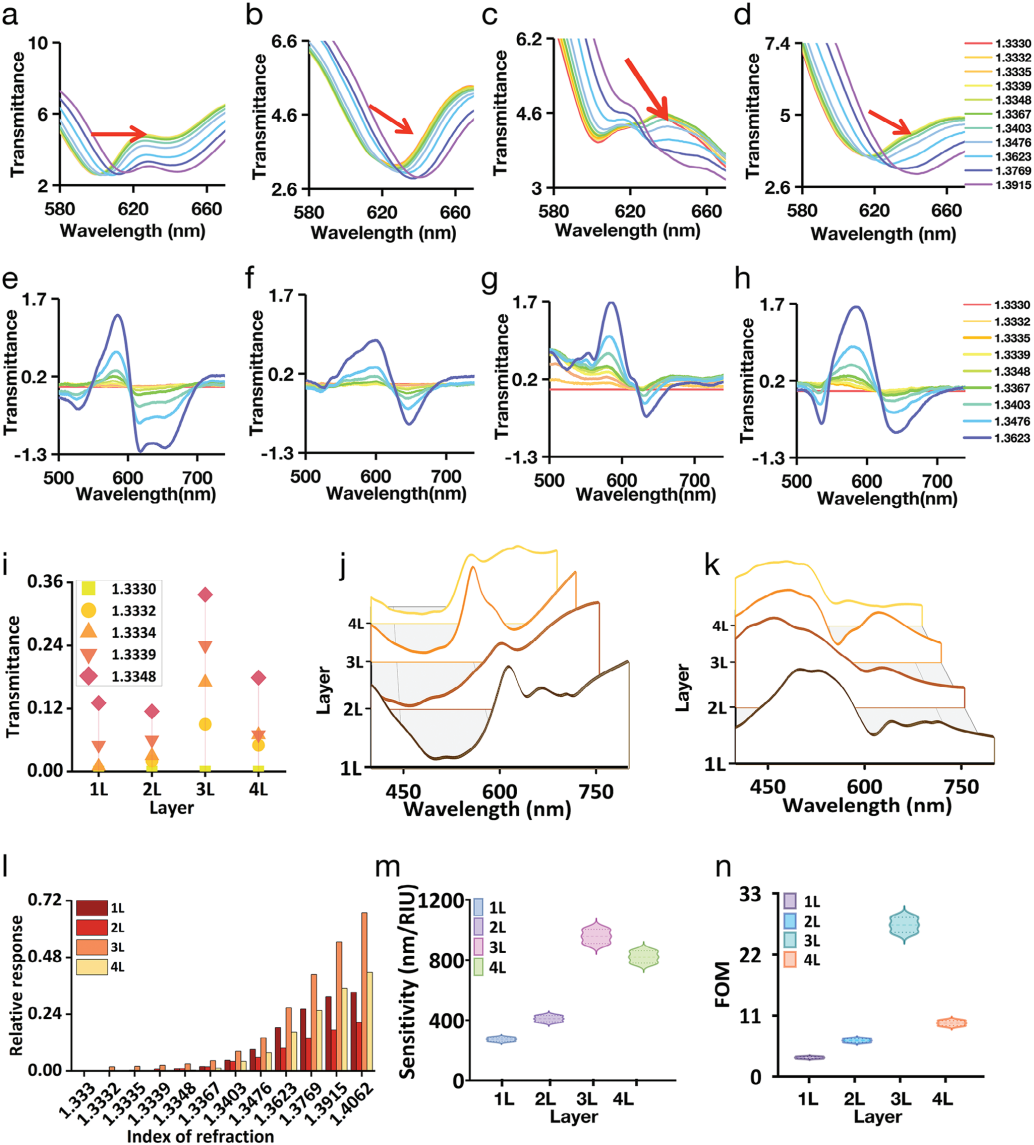
Sensitivity characterization of four types of MetaSPR chip sensors by measuring different concentrations of sucrose (0.15–40%), the position of the characteristic resonance peak is locally enlarged, and the full spectrum is shown in Figure S6 (Supporting Information). a–d) The transmittance changes of single‐layer, double‐layer, triple‐layer, and quadruple‐layer chips are detected using sucrose water under different refractive index changes. e–h) Differential spectroscopy of transmittance response signals for single‐layer, double‐layer, triple‐layer, quadruple‐layer chips. i) Dual‐wavelength differences between four chips. It could be observed that the triple layer has the highest response value. j) The comparison of the resonant peaks of the four types of chips in the air shows that the FWHM at the resonant peak of a triple‐layer chip is the narrowest. k) The comparison of the resonant peaks of the four types of chips in water. l) Dual‐wavelength differences in absorption values of sucrose aqueous solutions between four types of chips. m,n) Comparison of sensitivity and FOM of four metal chips.

The sensitivity of the four types of MetaSPR chips was characterized by measuring different concentrations of sucrose solutions (0–40%, corresponding to RI values of 1.3330–1.3915).^[^ ^14^ ^]^ The transmittance of these sucrose solutions was recorded using a transmission spectrometer. As the sucrose concentration increased (Figure S6, Supporting Information), the transmission spectrum demonstrated changes corresponding to the RI variations (**Figures** **3**a–d). By subtracting the transmittance of the sucrose solutions from that of water (Figures 3e–h), we observed changes in transmittance and wavelength shifts at ≈590 and 630 nm, respectively. These changes were observed for single‐, triple‐, and quadruple‐layer chips. In contrast, for a double‐layer chip, the observed wavelength shifts were ≈590 and 650 nm. Consequently, the transmittance response, which served as an output signal, was determined by subtracting the transmittance values at 630 nm from those at 590 nm, or 650 nm for the double‐layer chips. The two‐wavelength transmittance showed a positive correlation with the concentration of the sucrose solutions. Within the RI measurement range of 1.3330–1.3623 (corresponding 0–20% sucrose), the triple‐layer chip demonstrated the greatest signal change (Figure 3g), indicating its highest sensitivity. The multi‐layer free electron density chips, including double, triple, and quadruple‐layer configurations, all demonstrated high sensitivity, responding to transmittance variations as subtle as 0.15% sucrose (RI:1.3332). In contrast, the single‐layer chip only responded to variations of 0.3% sucrose (RI:1.3335) (Figure 3i). In addition, in terms of response values, the triple‐layer chip continued to respond to changes in sucrose at lower concentrations. Therefore, the resolution of the triple‐layer chips was ≈2–4 times higher than that of the single‐layer chip.

The performance of the MetaSPR sensor is typically evaluated using the FOM, which is defined as the ratio of the sensitivity to the width of the spectral peak (the full width at half maximum (FWHM) at the resonant peak).^[^ ^21^ ^]^ To compare the sensitivities of the four chips, we initially assessed the chips in two aspects: their RI and FOM calculated using Equation (2). The resonant peaks of the four types of chips were measured in air with a refractive index of 1.0003 (Figure 3j) and in water with a refractive index of 1.3330 (Figure 3k). We observed a shift in the peak wavelength with an increasing number of chip layers, with the FWHM of the triple‐layer chip being the narrowest. The transmittance of water and a 5% sucrose aqueous solution was measured to calculate the FOM and detection sensitivity (Table S1, Supporting Information) The detection sensitivity values for single‐layer to quadruple‐layer chips were 273 nm/RIU, 410 nm/RIU, 957 nm/RIU, and 820 nm/RIU, respectively (Figure 3m). Additionally, the FOM for single‐layer to quadruple‐layer chips were 3.42, 6.51, 27.34, and 9.65, respectively (Figure 3n). The triple‐layer chip had a sensitivity of 957 nm/RIU and FOM of 27.34, which are the highest values observed among the four types of chips tested. These values surpass those reported in previous studies.^[^ ^21^ ^]^ Notably, the detection sensitivity of the triple‐layer chip was 3.5 times greater than that of the single‐layer chip, and its FOM was 7.99 times higher, showcasing a significant enhancement in performance.

Our findings demonstrate that by manipulating the gradient of free electron density, the triple‐layer chip significantly enhances sensitivity. Fine‐tuning the thickness ratio of the silver‐to‐gold layers can further optimize performance, resulting in a narrower full width at FWHM.^[^ ^41a^ ^]^ This is consistent with the literature's emphasis on the importance of a narrow bandwidth for superior metamaterial design^[^ ^42^ ^]^ underscoring the pivotal role of material layering and thickness in achieving high sensitivity and selectivity in biosensing applications.

### 2.4 Quantitative Detection

**Figure 4:**
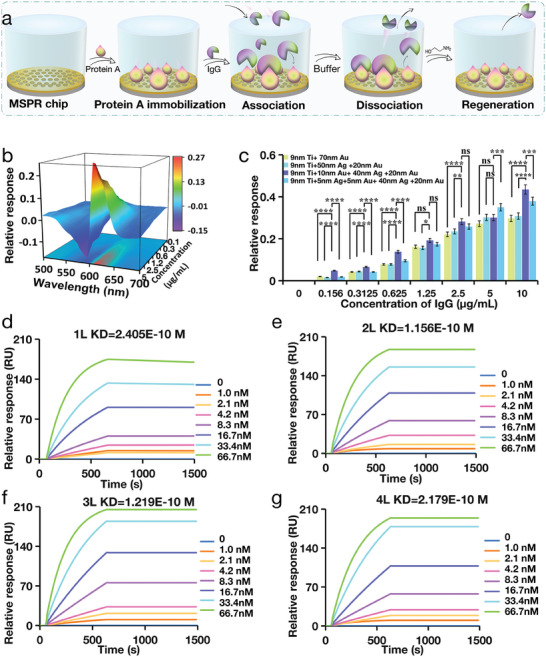
Quantitative analysis and affinity comparison of IgG. a) Process and schematic diagram of real‐time quantitative detection of IgG using multi‐layer free electron density MetaSPR chip to immobilize Protein A. b) Differential spectroscopy of IgG detection using triple‐layer chip immobilized Protein A. c) Quantitative analysis of the dual wavelength differences in IgG using four types of chips shows that the triple‐layer chip has the highest response value. Data are presented as mean ± standard deviation (SD) (n = 3). ^*^ *p* < 0.05, ^**^ *p* < 0.01, ^***^ *p* < 0.001 and ^****^ *p* < 0.0001. d–g) Real‐time detection data and affinity calculation results of the affinity between Protein A and IgG for single‐layer, double‐layer, triple‐layer, and quadruple‐layer chips, respectively. It can be found that the affinity results of the four chips are almost consistent, and the triple‐layer chip dynamic detection response value (RU) is also the largest.

Many factors influence the detection performance of the MetaSPR sensor.^[^ ^43^ ^]^ To assess the quantitative detection sensitivity of the four chips, we performed further evaluations using differential spectra and real‐time dynamic curves of Protein A against different concentrations of IgG antibodies. Protein A was immobilized onto the surfaces of the four different FED‐MSPR biosensors. Subsequently, we detected varying concentrations of IgG (0, 0.16, 0.31, 0.63, 1.25, 2.5, 5, and 10 µg mL^−1^) (**Figure** **4**a). In the typical differential absorption spectra and kinetic curves of the different chips, the dual‐wavelength optical density (OD) differential values of single‐ (OD_620_–OD_600_), double‐ (OD_615_–OD_595_), three‐ (OD_615_–OD_590_) (Figure 4b), and quadruple‐layer (OD_610_–OD_590_) chips (Figure S7a, Supporting Information), were proportional to the different IgG concentrations (Figure S7c, Supporting Information). The triple‐layer chip demonstrated the highest response value at each concentration tested. Specifically, at the highest concentration, the triple‐layer chip demonstrated a maximum reaction value of 0.435, showing the highest signal‐to‐noise ratio and improved detection sensitivity (Figure 4c; Figure S7b, Supporting Information).

### 2.5 Biokinetic Analysis

To further assess the detection performance of the four chips, dynamic analyses and comparisons were conducted utilizing molecular interaction platforms, focusing on the investigation of affinity changes between Protein A and IgG antibodies. Figure 4a shows the detection principle and process. Protein A was immobilized on the four‐chip sensor surface, and different concentrations of IgG (0–66.67 nM) were added to different sensor wells. Real‐time association dynamic curves were generated using a multifunctional molecular analyzer. After 10 min, the unbound liquid in the well was replaced with a buffer solution, and a dissociation curve was constructed for 15 min. A real‐time kinetic curve was obtained by fitting the continuously monitored relative response (RU) (Figure 4d).

The real‐time detection data and affinity calculation outcomes of Protein A and IgG affinities, derived from the dynamic analysis of single, double, three, and quadruple‐layer chip sensors, indicated that the KD values were as follows: 2.405E‐10 M for the single‐layer, 1.156E ‐10 M for the double‐layer, 1.219E ‐10  M for the triple‐layer, and 2.179E ‐10 M for the quadruple‐layer (Figures 4d–g). Significantly, the affinity results of the four chips were largely consistent and corresponded with the reported affinity of 2.30E ‐10 M.^[^ ^44^ ^]^ Compared with their dynamic parameters and response values, the KD values of the multi‐layer FED‐MSPR chips showed reasonable consistency with those of the single‐layer metal chips, with the triple‐layer FED‐MSPR chips exhibiting the highest response values. This indicates that the triple‐layer chip is more conducive to the collection of response signals, offering a stronger signal‐to‐noise ratio. The differences in the KD calculation results among the four chips are not significant, indicating that triple‐layer chips can still have the same detection performance as pure gold chips while saving costs and improving sensitivity. Consequently, they can serve as viable alternatives to traditional single‐layer gold chips as consumables for molecular interaction instruments and have broad application prospects.

Therefore, based on our comprehensive comparison of simulation outcomes, chip sensitivity, durability, quantitative detection results, and kinetic analyses, we conclude that the MetaSPR chip sensor, featuring materials with three distinct free electron density layers (Ti‐Au‐Ag‐Au), demonstrates optimal performance.

### 2.6 Mass Production and Characterization of the FED‐MSPR Chip

**Figure 5:**
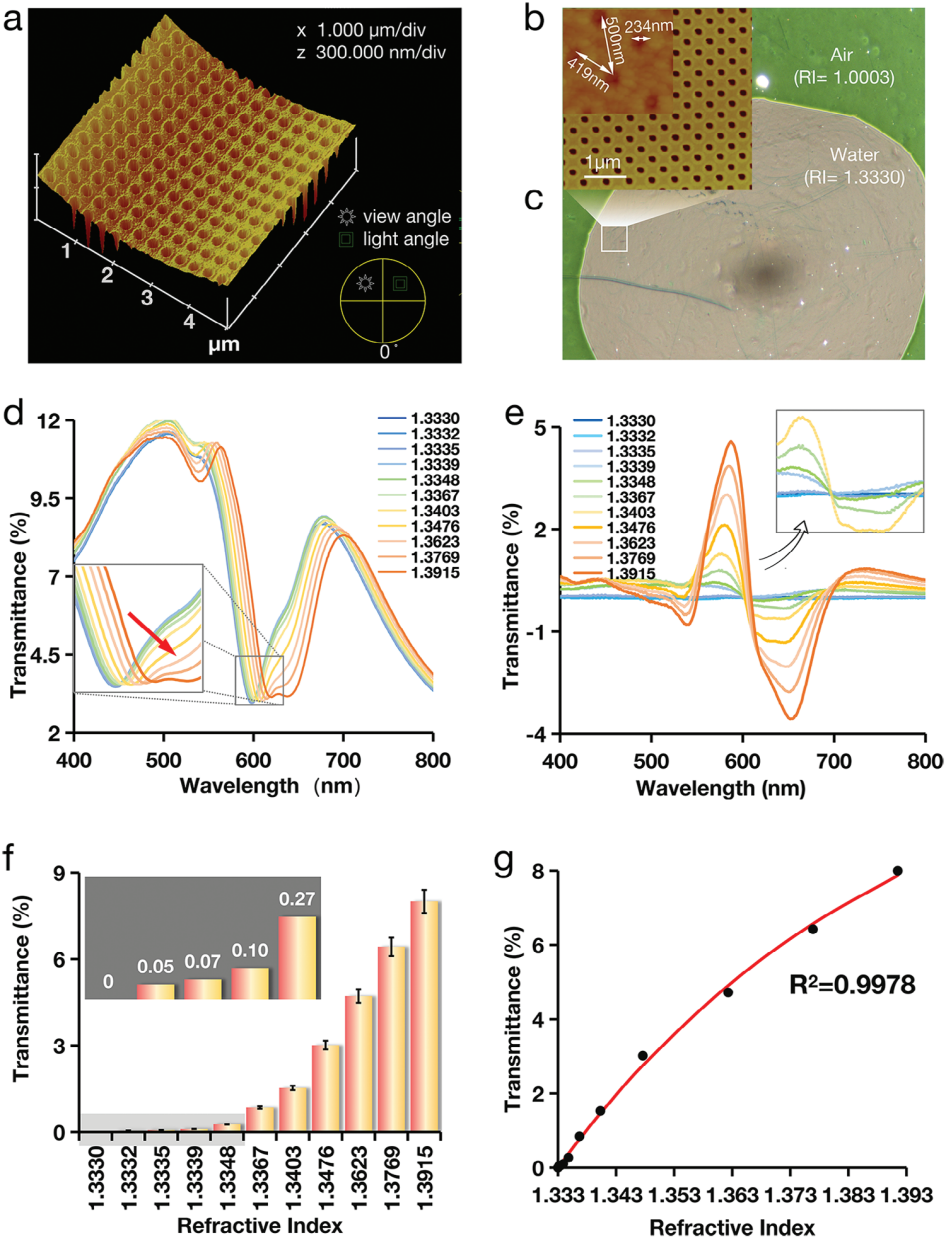
Characterization of the mass‐produced FED‐MSPR chip. a) Characterization of the 3D structure of the mass‐produced FED‐MSPR chip surface by AFM. b) AFM characterization planar view of nanocup array structure chip. c) The boundary line between air and water in a hole with a microscope water and air shows different colors (green and light brown, respectively). d) The full transmission spectra that converted by formula from the total absorption spectrum of sucrose at different concentrations (0–40%) with RI ranging from 1.3330 to 1.3915, with significant valley shift as the refractive index changes. e) Changes in the two‐wavelength transmittance of OD values conversion (OD _590_‐OD _630_ with different RIs. f) Histogram of two‐wavelength transmissivity showing changes at different RIs corresponding to varying concentrations of sucrose. Data are presented as mean ± standard deviation (SD) (n = 3). g) There exists a correlation between changes in transmittance and refractive index.

After identifying the most effective FED‐MSPR chip, we proceeded to mass‐produce this sensor material while maintaining rigorous scientific and technological characterization and testing. The transition to mass production involved refining our manufacturing processes while preserving the chip's structural integrity and performance, as evidenced by AFM analysis revealed a high level of uniformity across the nanocups array (**Figures** **5**a,b). The sensor chip surface showed distinct colors in media with different RI, including air (RI = 1.0003) and water (RI = 1.3333), indicating superior detection sensitivity (Figure 5c).

To further assess the sensitivity of the mass‐produced FED‐MSPR chip, we subjected it to varying concentrations of sucrose solutions and analyzed the absorption spectra The absorption value was converted into transmittance using Equation (3). The resulting transmission spectra demonstrate a downward gradient shift toward the right as the RI of the sucrose solution increases, indicative of a redshift in the resonance peak (Figure 5d). By subtracting the transmittance of water from that of the sucrose solutions (Figure 5e), we normalize the spectra to highlight changes in light intensity at specific wavelengths. Consequently, by comparing the dual‐wavelength differences values (OD_630−_OD_590_), the FED‐MSPR chip demonstrated remarkable sensitivity, detecting a sucrose change of 0.15% (Figure 5f). This represents a two‐fold enhancement over the concentration of sucrose (0.3%) previously reported as responsive to a double‐layer gold‐silver chip.^[^ ^15b^ ^]^ The relationship between the concentration of the sucrose solutions and the dual‐wavelength differences values demonstrated a clear positive correlation. This correlation was precisely quantified through logistic fitting, which resulted in a correlation coefficient (R^2^) of 0.9978 (Figure 5g). In addition, using the automated sampling molecular interaction instrument (WeSPR One, XLEMENT), we were able to observe in real‐time that the mass‐produced FED‐MSPR chip's baseline consistently returned to its original state before and after regeneration (Figure S8, Supporting Information), confirming its durability and repeatability. These indicated that the mass‐produced FED‐MSPR chip demonstrated an excellent detection performance.

The mass‐produced FED‐MSPR chip can be seamlessly integrated with 96‐well and 384‐well assay platforms commonly used in molecular interaction studies for automated high‐throughput assay systems. By using cost‐effective silver material, compared to a single‐layer gold chip, we have saved two times the production cost per 12‐inch wafer. This not only reflects our commitment to cost efficiency but also supports the sustainable adoption of our technology. In addition, our manufacturing methodology ensures the production of at least 70 96‐well or 384‐well MetaSPR sensors per day, highlighting the readiness of the technology for large‐scale molecular screening applications.

### 2.7 Affinity Assessment of Immunotherapy Drugs based on the Multifunctional FED‐MSPR Biosensor

SPR is widely used in pharmacokinetic drug profiling^[^ ^45^ ^]^ and high‐throughput screening.^[^ ^46^ ^]^ This technology provides significant insights into interaction rates and binding levels, enabling the determination of kinetic constants. Such data are invaluable for the strategic design of new therapeutic molecules and for distinguishing therapeutic differences among similar drug compounds.^[^ ^35^ ^]^ MetaSPR technology, an advancement of SPR, was also designed for label‐free kinetic analyses. To demonstrate its multifunctional capabilities, we utilized the triple‐layer FED‐MSPR biosensor for the affinity assessment of the immunotherapeutic drug (Adalimumab) and its target protein (TNF‐α). Adalimumab plays an essential role in inhibiting the significant inflammatory effects induced by TNF‐α, such as endothelial activation, endothelial monocyte adhesion, and endothelial leakage. This extends the therapeutic potential of Adalimumab in managing autoimmune disease.^[^ ^47^ ^]^ Consequently, the detection of Adalimumab drugs holds immense practical significance in clinical settings.

**Figure 6:**
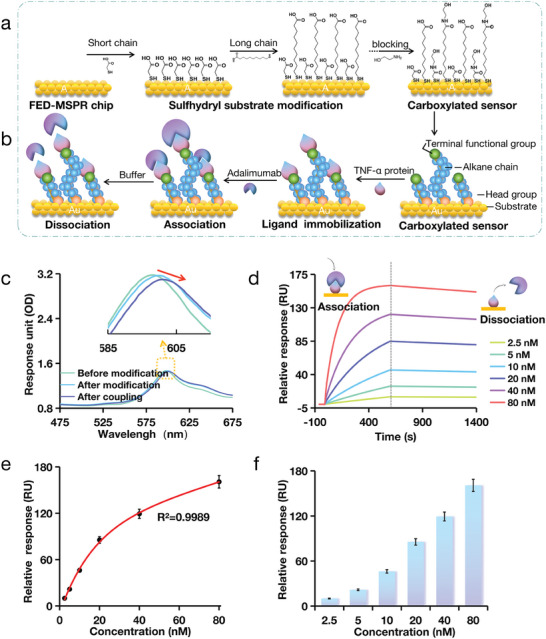
The affinity assessment results of Adalimumab drugs using the FED‐MSPR biosensor. a) Carboxylation process of the FED‐MSPR biosensor. b) The process of antibody affinity assessment using carboxylation FED‐MSPR biosensor immobilized protein. c) Displacement changes in key steps during chip modification and sensor fabrication. d) The real‐time assessment data of the affinity between TNF‐α protein and Adalimumab. e) Adalimumab standard curve using the TNF‐α protein chip sensor (R^2^ = 0.9989). Data are presented as mean ± standard deviation (SD) (n = 3). f) The relative response of Adalimumab at different concentrations in the kinetic detection data. Data are presented as mean ± standard deviation (SD) (n = 3).

The 2D modification primarily involves the carboxylation of chips using thiol reagents, such as self‐assembled monolayers (SAMs). SAMs consist of three components: head, alkyl, and terminal functional groups. In this study, we selected short‐chain thioglycolic acid and long‐chain MUA, mixing them at a 75% ratio and incubating them step‐by‐step on the FED‐MSPR chip surface (**Figure** **6**a). These molecules bind to the surface of the nanocup array structure chip through the Au─S bond of the head group. The alkyl chains form a dense layer on the chain surface, and the long (─CH2─) chains demonstrate strong interactions, ensuring a high density of SAMs. The active groups at the end provide surface chemical functionalities such as the immobilization of antibodies and proteins. Following the 2D chemical modification of the FED‐MSPR chip, excess sites with functionalized end groups were sealed using low‐concentration ethanolamine reagents (Figure 6b). Each modification step resulted in a rightward shift in the absorption spectrum, indicating that a chemical substance bound to the surface of the chip (Figure 6c) caused a change in the resonance peak. SEM can be observed that after modification, the nanocup array structure on the chip surface is evenly distributed (Figure S9a, Supporting Information). Energy‐dispersive X‐ray Spectroscopy (EDS) analysis of the carboxylated chip surface shows that the introduction of carboxyl groups has led to discernible elemental energy distributions in the EDS spectrum (Figures S9b,c, Supporting Information). These findings collectively demonstrate that carboxyl functional groups were successfully grafted onto the FED‐MSPR chip surface. At this self‐assembly density, the carboxyl functional groups are sufficient to completely cover the bare gold surface, enhancing surface properties that are conducive to subsequent biomolecular interactions. Hydroxyl groups do not bind to the inactive hydroxyl or amino groups. Therefore, arranging the COOH and OH sequences at the end groups can effectively reduce non‐specific reactions.

After the 2D chemical modification of the FED‐MSPR chip, TNF‐α protein was synthesized immobilized on the FED‐MSPR biosensor surface by the amide condensation method. Different concentrations of Adalimumab were added to different wells, and real‐time dynamic profiles were monitored. Following 10 min of association, the unbound liquid was replaced with a buffer solution to create a 15 min dissociation curve. The dynamic curves were obtained by continuously fitting the relative RU values (Figure 6d). Linear calculation revealed a positive correlation between the relative response and the detection concentration, with a linear correlation coefficient of 0.9989 (Figure 6e‐f). The affinity data were fitted according to Equations (4 and 5), and the results of the fit were calculated. The association rate constant (Ka) of the TNF‐α protein to Adalimumab was 6.70E+5 M^−1^ s^−1^, and the dissociation rate constant (Kd) was 4.60E–4 s^−1^, giving the dissociation equilibrium constant KD of 6.86E–10 M. This affinity result is consistent with the data published on the official website of the raw material manufacturer. On their platform, they utilized the CM5 chip with Biacore, yielding an affinity of 2.55E–10 M (Figure S10, Supporting Information). To verify the accuracy of these test results, an SPR assay was performed using Biacore T200 to compare the differences between the SPR and MetaSPR assay results. The test results revealed the KD of 7.73E–10 M (Figure S11 and Table S2, Supporting Information). These values fell within an acceptable range, aligning closely with our test results. The affinities of the MetaSPR (WeSPR200) and SPR (Biacore T200) assays were almost identical. This demonstrates the accuracy of the affinity assessment results based on the FED‐MSPR biosensor. Our study underscores the versatility of the FED‐MSPR biosensor. Therefore, our novel FED‐MSPR biosensor could find wide applicability in drug detection and kinetic analysis. It offers a label‐free and real‐time method for assessing the affinity of immunotherapeutic drugs, thereby contributing significantly to drug development with substantial practical implications.

## 3 Conclusion

This study proposes a novel FED‐MSPR (Ti‐Au‐Ag‐Au) biosensor, based on a nanocup array structure PET film for affinity assessments in various stages of drug discovery, screening, and efficacy evaluation. The sensor's exceptional sensitivity and label‐free detection capability offer significant advancements in the efficiency of drug candidate screening and the precision of efficacy evaluations, potentially accelerating the drug development. The result demonstrates that the multilayer FED‐MSPR chip, particularly the triple‐layer variant, provides superior sensitivity and FOM compared to single‐layer gold chips. The triple‐layer chip, in particular, showed a substantial increase in sensitivity (3.5 times) and FOM (7.99 times), indicating its potential for detecting lower concentrations of biomolecular interactions. Furthermore, the comparative analysis of the response to RI variations and the detection of IgG demonstrated that the triple‐layer chip outperformed others in both absorption and transmittance, suggesting a superior detection threshold—a significant finding. The assessment of the FED‐MSPR platform, focusing on stability, sensitivity, and biokinetic analysis, confirmed its effectiveness in practical biomolecular interaction applications. Furthermore, the cost‐effective design of our FED‐MSPR biosensor, utilizing Ag and Au, suggests a viable alternative to conventional single‐layer MetaSPR sensors. This positions our technology as a promising tool for practical applications in life sciences and drug development. Based on this research, it was demonstrated that by regulating the free electron density gradient of a variety of metal layers on the chip surface, a biosensor with higher detection sensitivity may be achieved.

In addition, the mass‐produced FED‐MSPR chip's surface, modified with high and low staggered carboxylation, serves as an effective affinity assessment platform for evaluating the metabolizing ability of TNF‐α protein with the Adalimumab drug in vitro. This platform is instrumental in assessing drug activity against autoimmune diseases, contributing to the development of monoclonal antibody therapies. Looking ahead, the demonstrated potential of our biosensor in detecting immunotherapeutic drugs opens avenues for future research. We foresee continued innovation in the development of biosensors with free electron density gradients, expanding their utility in targeted drug screening and personalized medicine.

## 4 Experimental Section

### Materials

Hexylsilane, 6‐mercapto‐1‐hexanol (MCH), thioglycollic acid, 11‐mercaptuoudecanoic acid (MUA), 1‐ethyl‐3‐(dimethylaminopropyl) carbodiimide (EDC), N‐hydroxysuccinimide (NHS), bovine serum albumin (BSA), ethanolamine and phosphate‐buffered saline (PBS) buffer, 2‐ (N‐morphinyl) ethanesulfonic acid (MES) solution, gelatin from cold water fish skin, and casein were purchased from Sigma–Aldrich (Shanghai, China). Recombinant Protein A (Cat:10600‐P07E) was purchased from Sino Biological (Beijing, China). Immunoglobulin Antigen (Human IgG, Cat: No. SP001) was purchased from Solarbio (Beijing, China). Adalimumab (Cat.: HY‐P9908) was purchased from Med Chem Express (Shanghai, China). Human TNF‐alpha protein (TNF‐α, Cat.: TNA‐H4211) was purchased from ACRO (Beijing, China). All chemicals were used without further purification. PET film was purchased from Yixing Sunshine Plastic Co., Ltd. (Yixing, China). Atomic force microscope (AFM), model: Veeco Nano Scope Multi Mode, was tested on the micro nano platform of Huazhong University of Science and Technology (Wuhan, China). An XLement absorbance multifunctional analyzer (model: WeSPR200) was used to read the optical density (OD) values (Shanghai, China).

### Fabrication and Characterization of the Nanocup Array Structure MetaSPR Sensor Chip

The evaporation plating of the MetaSPR chip was based on previous research within the same research group.^[^ ^22^, ^48^ ^]^ A replica molding technique was used for fabrication, with adjustments made to optimize the production process. The specific production process is outlined in Figure S12 (Supporting Information). Initially, a mold with a nano‐cone pattern was created using laser interference lithography on a quartz substrate. The original mold featured a periodic tapered nanopillar array with an upper diameter, lower diameter, and height of 100, 200, and 500 nm, respectively. The mold was subjected to surface treatment with silane to achieve hydrophobicity. Subsequently, UV curing glue was uniformly applied to the mold, and the PET film was fitted. UV light (105 mW cm^−2^) was used for curing for 45 s. Subsequently, the PET substrate with a periodic nanohole pattern was carefully separated from the mold, forming a polymeric nanocup array structure. Furthermore, heavy metals with plasmon resonance effects were deposited onto the polymeric nanocups array using an electron beam evaporator. The evaporation parameters of the metal layers were configured as follows: single (9 nm Ti and 70 nm Au), double (9 nm Ti,50 nm Ag, and 20 nm Au), triple (9 nm Ti, 10 nm Au, 40 nm Ag, and 20 nm Au), and quadruple (9 nm Ti, 5 nm Ag, 5 nm Au, 40 nm Ag, and 20 nm Au) layers. The resulting nanocups array MetaSPR sensing chips were prepared, followed by cutting them into 13 cm × 8.5 cm sections, affixing them onto the open‐bottom 96‐well plate, and assembling them into the 96‐well chip sensor configuration.

### Experimental Methods for Stability Analysis

The morphologies of all chips were examined using AFM. Initially, the chips were placed in a culture dish and stirred at 37 °C and 700 rpm for 24 h. Subsequently, they were thoroughly washed with ddH_2_O and dried with nitrogen. The chip surfaces were then scrutinized for damages using a microscope. The chip surfaces were scanned using AFM, with a scanning size and rate set to 5 µm and 1.00 Hz, respectively. Any changes in the surface roughness of the chips before and after stirring in PBS were compared.

### Experimental Method for Analyzing Chip Sensing Performance

The sucrose aqueous solution with concentrations of 0–40% were prepared using a mass‐to‐volume ratio to verify the sensitivity of the chip in response to changes in surface substances. Subsequently, 50 µL of this solution was added to each well of the biosensor chips. The absorption spectra were measured at the full wavelength (500–700 nm) using a universal enzyme marker. Additionally, transmittance spectra were obtained using a transmission spectrometer.

Water (RI = 1.3330) and 5% sucrose (RI = 1.3404) were added to the MetaSPR chip device, and the optical response and sensitivity were monitored using a universal absorbance spectrum analyzer. The specific calculation process and methodology are in the Section 4 of Supporting Information. The FOM of the MetaSPR optical chip was comprehensively characterized using the following formula:

(2) $$FOM=\frac{Sensitivity}{FWHM\;}\;$$

After identifying the MetaSPR chip with the best performance, the uniformity of the chip was evaluated using AFM. The displacement of the chip in response to changes in the RI of sucrose water was measured, and the absorbance values were converted to transmittance using the following formula:

(3) A=−logT

### Biodynamic Analysis Approach to Biosensors

After the chip was cleaned, Protein A was diluted to 20 µg mL^−1^ with CBS buffer; 2 µL/well was immobilized on the surface of the MetaSPR chip for 16 h. Subsequently, the chip surface was treated with a 1% BSA solution for 30 min at 37 °C to prevent nonspecific binding. For the affinity assays, 50 µL of IgG analytes (ranging from 0.1 to 66.7 nM) in PBS buffer were simultaneously introduced into the chip wells. The binding process were monitored for 10 min. After the association period, 150 µL of PBS buffer were added to the chip wells, and the dissociation kinetic curve was rapidly monitored for 15 min.

### Surface Functionalization of the MetaSPR Biosensor

The MetaSPR chip was cleaned with 50 mM NaOH solution, 50 mM HCl solution, 50% ethanol solution, and ddH_2_O sequentially before any chemical modification and dried with nitrogen gas. Subsequently, 50 µL of 1 mM thioglycolic acid was added to each well and allowed to react at 37 °C for 2 h. After the reaction was complete, the chip‐hole plate was washed alternately three times with 75% alcohol and water. Similarly, 50 µL of 1.3 mM MUA was added to each well and incubated for 2 h at 37 °C. The cleaning steps were then repeated. After fabrication of the carboxylation biosensor, 30 µL of NHS solution (50 mM, pH 6.0 MES) was added to the chip well, followed by the addition of 30 µL EDC (50 mM, pH 6.0 MES). The mixture was gently patted to ensure thorough mixing, and the chip plate was then incubated at 37 °C for 30 min. After completion of the reaction, the chip was washed thoroughly and dried. Subsequently, the TNF‐α protein was diluted and dissolved to 25 µg mL^−1^ with MES solution at pH 4.5. A 2 µL aliquot of the ligands was added to the center of each well and covalently coupled and immobilized at 4 °C for 16 h. Then, PBS buffer containing 1 M ethanolamine (150 µL) was added into each well to block the unbound carboxyl groups at 37 °C for 15 min. Subsequently, PBS buffer containing 0.05% fish gelatin and 0.5% casein (150 µL) was added to block the non‐specific adsorption site of the MetaSPR sensor at 37 °C for 1 h. Finally, the modified chips were sealed with sealing membranes and stored at 4 °C for subsequent analysis.

### Affinity Assessment for the TNF‐α Protein and Adamulimab

Establishment of the MetaSPR carboxylation biosensor using the direct binding method for kinetic measurements: Before conducting the affinity assay, the sensor well of the MetaSPR chip was rinsed and stabilized with HBS‐ET buffer. Subsequently, HBS‐ET buffer containing different concentrations of adamulimab was added to the sensor well to assess the binding of adamulimab to the TNF‐α protein immobilized in the well. The interactions in the wells were monitored using an analyzer during the binding and dissociation periods of 10 and 15 min, respectively. Following each binding cycle, a 10 mM glycine solution was used to regenerate the surface of the MetaSPR chip. Finally, PBST buffer containing 5% sucrose was added to the utilized chip, as a protective solution. The plate was subjected to oven‐drying and stored at 4 °C for subsequent reuse.

The fitting formula for the association dynamic analysis was selected from Origin Graph software, BoxLucas1 was chosen, the formula as follows:

(4) $$y=a\;*\left(1-\exp\left(-b\;*\;x\right)\right)$$

The fitting formula for the dissociation curve was chosen Exp2PMod1 was chosen, as follows:

(5) $$y=a\;*\;\exp\left(b\;*\;x\right)$$

### Statistical Analysis

The MetaSPR data were analyzed using Origin 2021 software. Significant difference used GraphPad Prism 9.5.1 software. All data was presented as mean standard deviation (SD). The statistical comparison between groups was performed following the student's t‐test (two‐tailed). ^*^ *p* < 0.05, ^**^ *p* < 0.01, ^***^ *p* < 0.001 and ^****^ *p* < 0.0001. Results with *p* < 0.05 were considered statistically significant.

## Conflict of Interest

The authors declare no conflict of interest.

## Supporting information



Supporting Information

## Data Availability

The data that support the findings of this study are available from the corresponding author upon reasonable request.
