# Supplementary material for: Free Electron Density Gradients Enhanced Biosensor for Ultrasensitive and Accurate Affinity Assessment of the Immunotherapy Drugs
Source: Adv Sci (Weinh). 2024 Oct 23;11(46):2404559. doi: 10.1002/advs.202404559 (PMC11633510; doi:10.1002/advs.202404559)
Supplement: Supplementary file 1 — Supporting Information [file ADVS-11-2404559-s001.docx]

Supporting Information

**Free Electron Density Gradients Enhanced Biosensor for Ultrasensitive and Accurate Affinity Assessment of the Immunotherapy Drugs**

Youqian Chen^1^, Hongli Fan^1^, Rui Li^1^, Huazhi Zhang^2^, Rui Zhou^2^, and Gang L. Liu^1,^*, Chunmeng Sun^3,4,*^, Liping Huang^1,2,5^*

^1^College of Life Science and Technology, Huazhong University of Science and Technology, 1037 Luo Yu Road, Wuhan 430074, P. R. China.

^2^Biosensor R&D Department, Liangzhun (Wuhan) Life Technology Co., Ltd., Wuhan 430070, China.

^3^Department of Pharmaceutics, School of Pharmacy, China Pharmaceutical University, 639 Longmian Avenue, Nanjing 211198, China

^4^NMPA Key Laboratory for Research and Evaluation of Pharmaceutical Preparations and Excipients, China Pharmaceutical University, 24 Tong Jia Xiang, Nanjing 210009, China

^5^School of Food Science and Pharmaceutical Engineering, Nanjing Normal University, Nanjing 210023, PR. China

*Corresponding authors

E-mail: lphuang@aliyun.com (L. Huang); [suncmpharm@cpu.edu.cn](mailto:suncmpharm@cpu.edu.cn) (C. Sun); [loganliu@hust.edu.cn](mailto:loganliu@hust.edu.cn) (Gang L. Liu)

**1 Simulation of the Enhancement Effect of Free Electron Density Gradient.**

3D finite-difference time-domain (FDTD) simulations were conducted utilizing the commercial software FDTD Solutions (Lumerical Inc., Vancouver, BC, Canada). The model replicated the geometry of the sensor device, featuring a periodicity of 400 nm, a nanocup top diameter of 200 nm, and a nanocup height of 500 nm. The background refractive index was established at 1.333 to emulate a liquid environment. The sensor chips were subjected to illumination by a plane wave from the top side (-z direction). A perfect matching layer was implemented for the boundary conditions in the Z-axis, while periodicity was enforced for the boundary conditions along the X- and Y-axes. ^[1]^ .Utilizing 3D-FDTD software, simulations were conducted to analyze the electric field and transmittance characteristics of four distinct types of chips. The results indicate minimal variance in electric field activity among the four chips at the resonance peak (Figure S1), yet a notable disparity in electric field distribution was observed at the valley. The discrepancy of 5-10nm between the simulated full spectrum transmission and the experimentally detected wavelength, as illustrated in Figure S1a-d, suggests minimal variations in simulated transmittance across the four chips. These spectral disparities may be attributed to factors including the wettability of the device.^[2]^


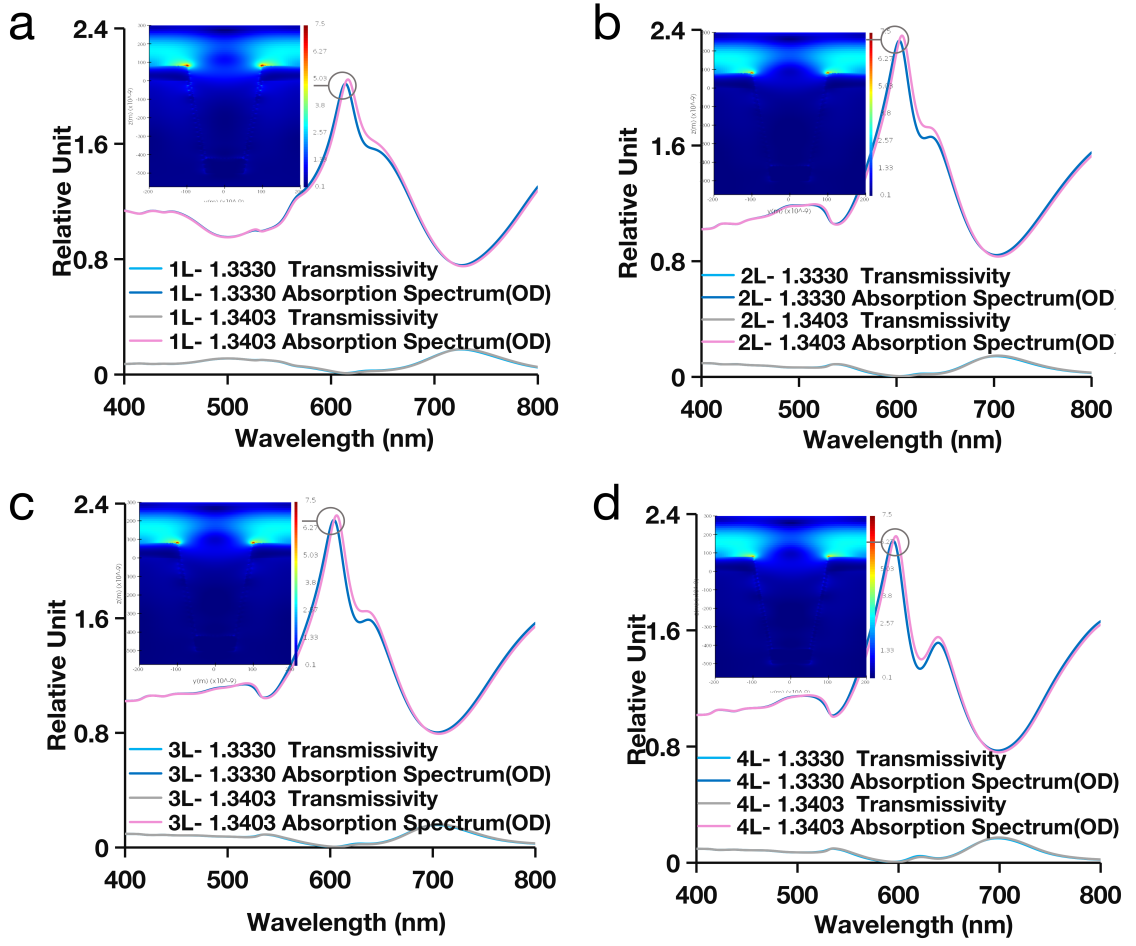


**Figure S1** Presents the simulation results of 3D-FDTD. Figure a-d respectively illustrate the full transmission spectrum and absorption spectrum of 1L, 2L, 3L, and 4L chips simulated by FDTD, as well as the simulated free electric field at the resonance peak of these chips.


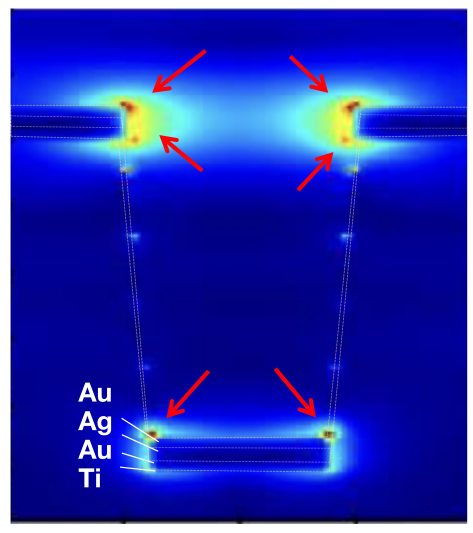


**Figure S2** Illustrates the most active free electric field distribution region for sensors with varying combinations of electron densities.

**2 Stability of chips**

We examined chip performance across a range of environmental conditions. Agitated in water solutions with pH 4.5, 6.0, 7.5 and 9.0 at 300rpm for 48 hours, examination showed no surface degradation across all pH levels. Similarly, immersion in water at temperatures 4, 25, 37 and 42°C for 48 hours revealed no flaking or damage, indicating that temperature and pH have negligible effects on chip stability.


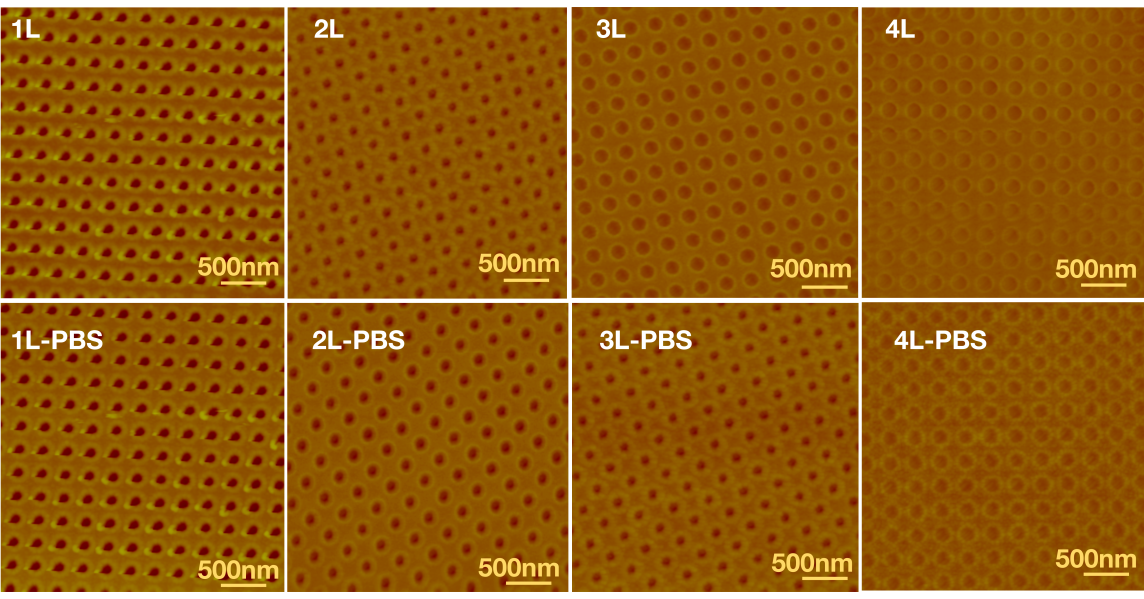


**Figure S3** AFM characterization of four types of chip surfaces. From left to right are the characterizations of single-layer (1L), double-layer (2L), triple-layer (3L), and quadruple-layer (4L) chips before and after shaking in PBS.


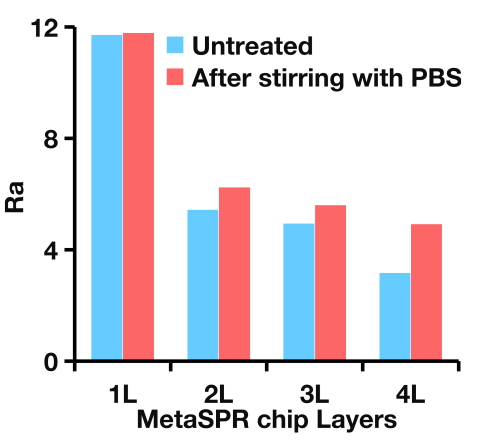


**Figure S4** Compares the surface roughness of four types of chips before and after PBS shaking.

**
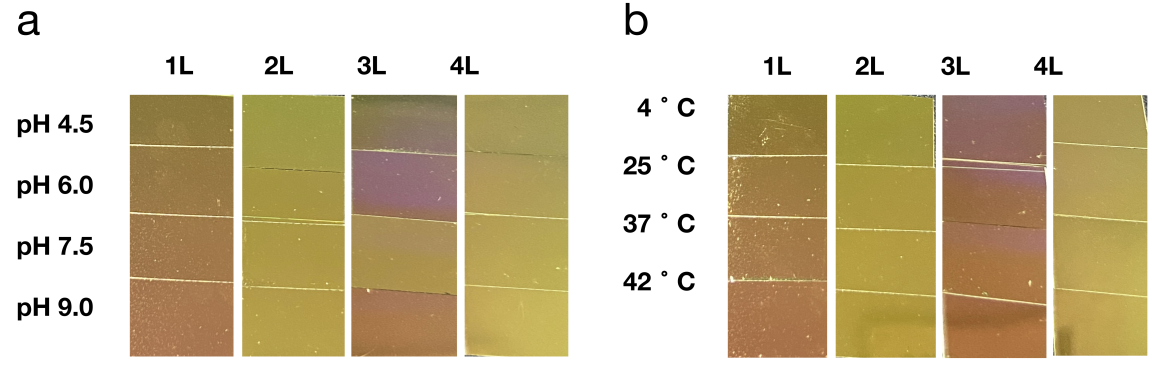
**

**Figure S5** (a) Comparison of surface morphology of four types of chips after soaking in solutions of different pH for 48 hours. (b) Comparison of surface morphology of four types of chips after soaking in solutions at different temperatures for 48 hours.

**3 Sensitivity Detection of Four Chips**

Utilizing a transmission spectrometer, the transmittance of four types of MetaSPR chips was characterized for sensitivity by measuring various concentrations of Sucrose (0.15%wt-40%wt). The full spectrum detection results are depicted in Figure S6, revealing a notable shift in the transmission spectrum with increasing refractive index (RI).


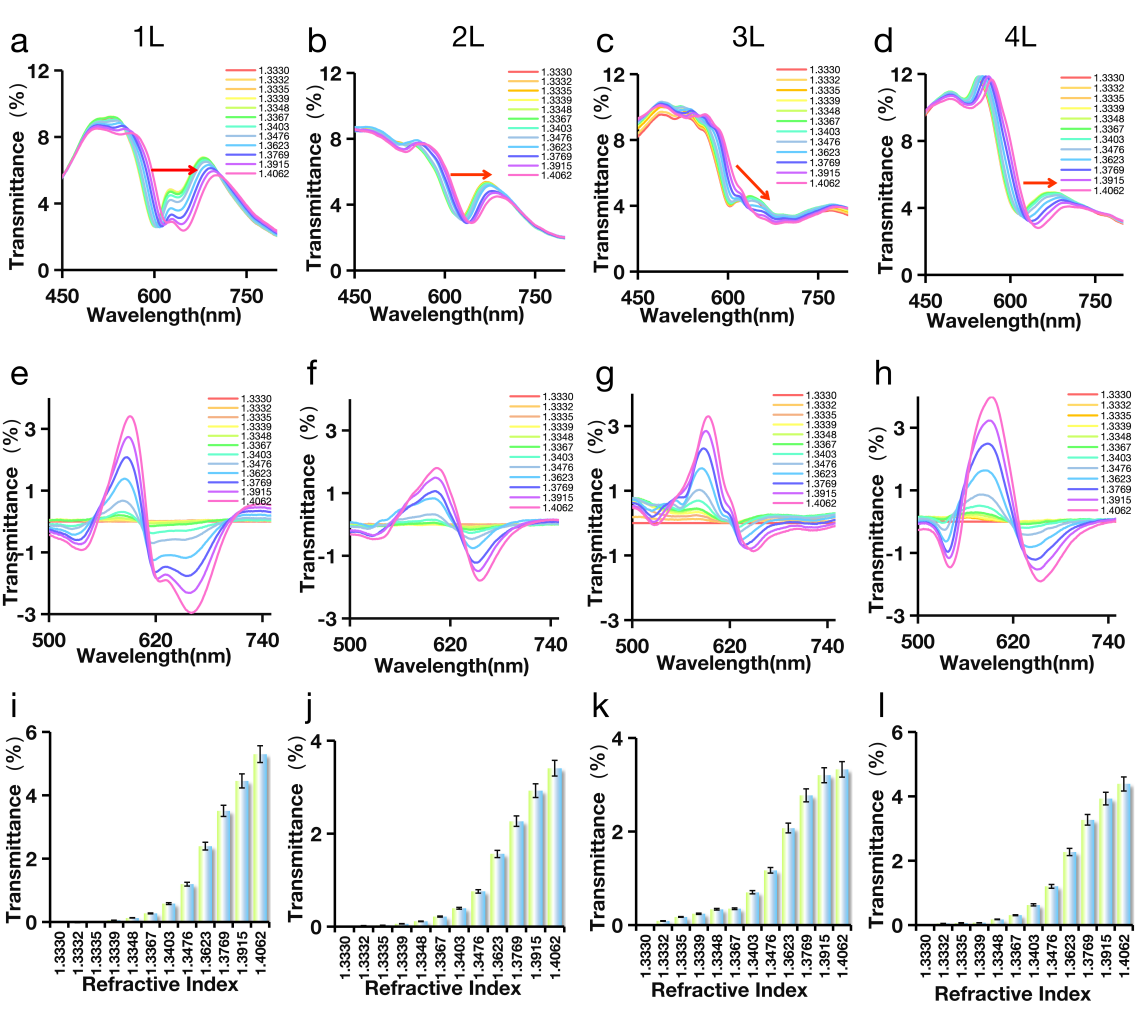


**Figure S6** Presents the sensitivity characterization of four types of MetaSPR chips through the measurement of various concentrations of Sucrose (0.15%wt-40%wt) . (a-d) The transmittance changes of 1L, 2L, 3L, and 4L chips were analyzed using sucrose water at different RI changes. The displacement of 3L chips becomes more pronounced as the refractive index increases. (e-h) Differential spectroscopy of transmittance response signals for 1L, 2L, 3L, and 4L chips revealed that the gradient differentiation of 3L chips is the most prominent, displaying the greatest variation.

**4 Performance testing methods and supplementary results**

The determination of sensor sensitivity is paramount in assessing the performance of a sensor. In our study, the MetaSPR sensor's performance is evaluated using the Figure of Merit (FOM), which is defined as the ratio of sensitivity to the spectral peak width at half maximum (FWHM) of the resonant peak.

**Sensitivity and FOM Calculation:**

The **sensitivity** is calculated using the formula:

$$Sensitivity=\frac{\lambda_{sucrose}-\lambda_{water}}{{RI}_{sucrose}-{RI}_{water}}$$

**FWHM Calculation Method:**

where λ_sucrose​_ and λ_water_ are the wavelengths corresponding to the highest original spectral peaks of 5% sucrose and water, respectively, and RI represents the refractive index.

The Full Width at Half Maximum (FWHM) of a spectral peak is calculated by first determining the optical density (OD) at the peak and at two distinct valleys within the spectral curve. The OD at the peak is denoted as OD_peak_, and the OD at the first and second valleys are denoted as ODvalley1 and ODvalley2, respectively. By subtracting ODvalley1​ from ODpeak, we obtain an intermediate OD value, OD1, which corresponds to a specific wavelength*λ*1 on the spectral curve. Similarly, subtracting ODvalley2 from ODpeak yields another intermediate OD value, OD2, corresponding to a different wavelength*λ*2. The FWHM is then defined as the difference between these two wavelengths, *λ*1 and *λ*2, effectively measuring the width of the peak at the OD levels that are intermediate to the peak and the valleys.

OD_peak_ be the optical density at the peak.

OD_valley1_ be the optical density at the first valley.

OD_valley2_ be the optical density at the first valley.

λ_1_ be the wavelength corresponding to OD_peak_ – OD_valley1_

λ_2_ be the wavelength corresponding to OD_peak_ – OD_valley2_

The FWHM can then be calculated using the following formula:

$$FWHM =\lambda_{1} - \lambda_{2}$$

**The FOM is then calculated as:**

$$FOM=\frac{Sensitivity}{FWHM}$$

**Table S1** Sensitivity and FOM values

| Layer | Displacement wavelength(nm) | FWHM | Sensitivity（nm/RIU） | FOM |
| --- | --- | --- | --- | --- |
| 1L | 2 | 80 | 273 | 3.42 |
| 2L | 3 | 63 | 410 | 6.51 |
| 3L | 7 | 35 | 957 | 27.34 |
| 4L | 6 | 85 | 820 | 9.65 |

**5 Quantitative Detection**

Four varieties of chips were employed for the immobilization of Protein A, followed by the detection of IgG antibodies. The absorption values of these chips were measured using a Microplate reader. The complete absorption spectra of the chips were illustrated in Figure S7a, showcasing distinct gradients in absorption across the four types, with the 3L chip exhibiting the most pronounced difference in spectral optical density. The results of dual wavelength subtraction (single- (OD_620_ – OD_600_), double- (OD_615_ – OD_595_), three- (OD_615_ – OD_590_), and quadruple-layer (OD_610_ – OD_590_) ) (Figure S7b) indicated that the 3L chip displayed the highest response value and sensitivity. There was an extremely strong linear relationship between the response values and IgG concentration for all four chips (Figure S7c).


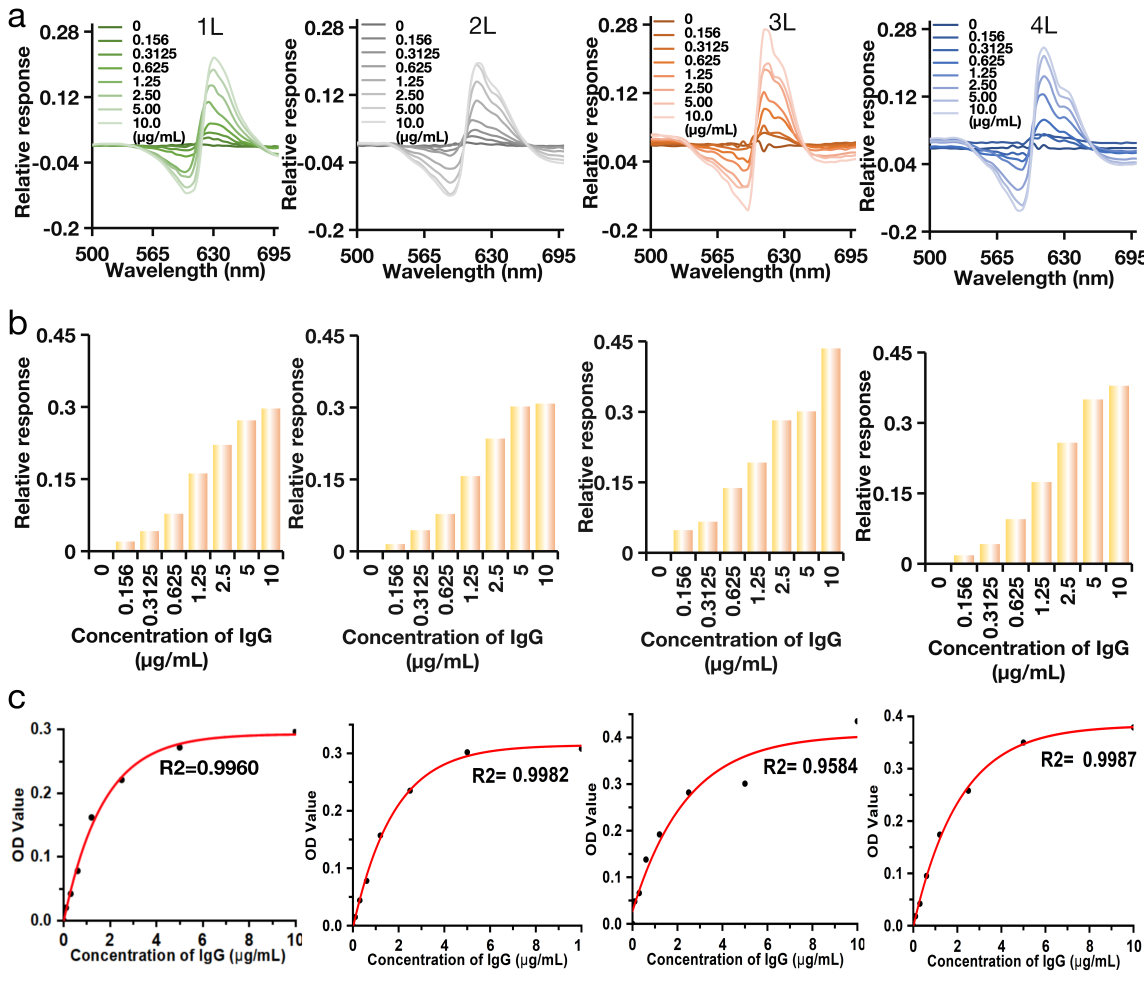


**Figure S7** Presents the quantitative detection results of IgG using four types of chips, including (a) the differential spectra, (b) dual wavelength differences, and (c) linear correlation coefficient between the response values and IgG concentration for each chip.

**6 The Calibration of FED-MSPR Biosensor**

The calibration of sensors is determined based on the regeneration effect. To provide a real-time visualization of the sensor's state before and after regeneration, Protein A was first immobilized on the sensor surface, followed by a blocking step, and then the sensor underwent continuous regeneration for IgG detection. The experimental findings indicate that upon completion of the regeneration process, the sensor's baseline was observed to revert to its original level, as depicted in Figure S8. That is, the sensor is capable of reverting to its pre-regeneration state following the regeneration process. The sensor has been calibrated and subsequent experiments can proceed normally.


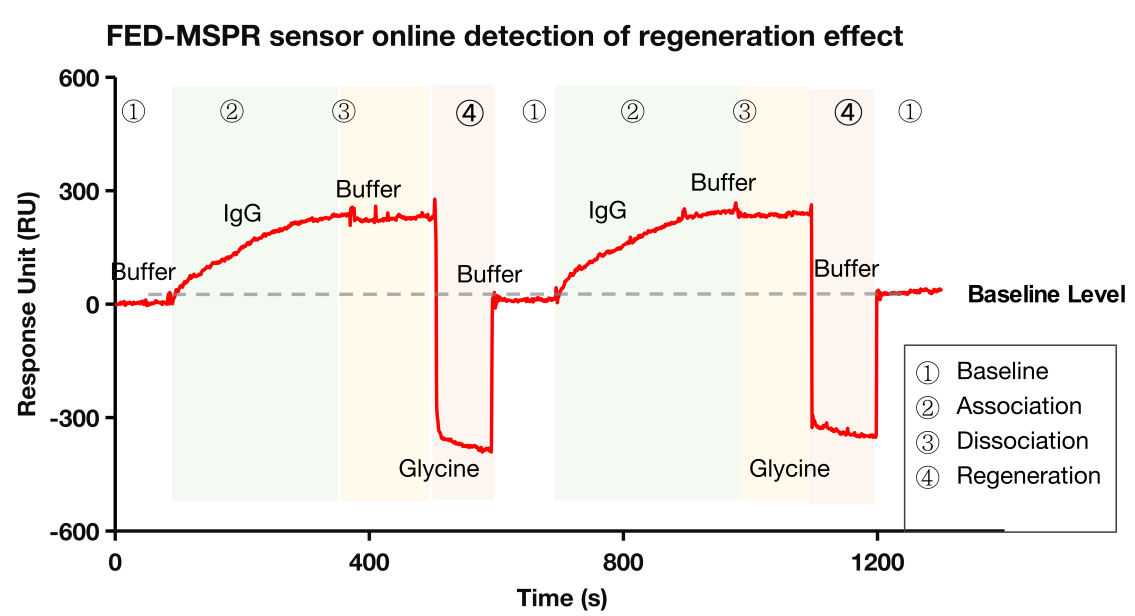


**Figure S8** Online testing of the regeneration effect of the FED-MSPR sensor showed that the regenerated baseline returned to the pre-regeneration baseline level.

**7 Characterization of Modified the MetaSPR Chip**

SEM can be observed that after modification, the nanocup array structure on the chip surface are evenly distributed (Figure S9a). Energy-dispersive X-ray Spectroscopy (EDS) analysis of the carboxylated chip surface shows that the introduction of carboxyl groups has led to discernible elemental energy distributions in the EDS spectrum (Figure S9b-S9c). These findings collectively demonstrate that carboxyl functional groups were successfully grafted onto the FED-MSPR chip surface. At this self-assembly density, the carboxyl functional groups are sufficient to completely cover the bare gold surface, enhancing surface properties that are conducive to subsequent biomolecular interactions.


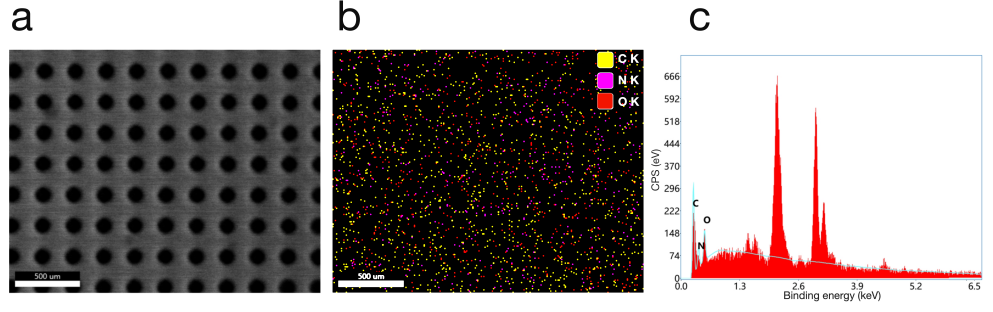


**Figure S9** (a) SEM image of the modified FED-MSPR chip. (b)EDS analysis characterizing the elemental composition and distribution on the surface of the MLM-Chitosan chip. (c) Elemental distribution map of the FED-MSPR biosensor chip, as determined by EDS.

**8 Comparison results of affinity detected on different platforms**

The results of the surface plasmon resonance (SPR) affinity test for Adalimumab and TNF-α were published on the official website of the manufacturer of the raw materials (Figure S10). According to the reports on the website, the interaction between Humira (Adalimumab) immobilized on a CM5 chip via anti-human IgG Fc antibodies and Human TNF-alpha, premium grade (Cat. No. TNA-H4211), was found to have an affinity constant of 0.255 nM (2.55E-10 M) as determined in a SPR assay using a Biacore T200 instrument (Routinely tested).


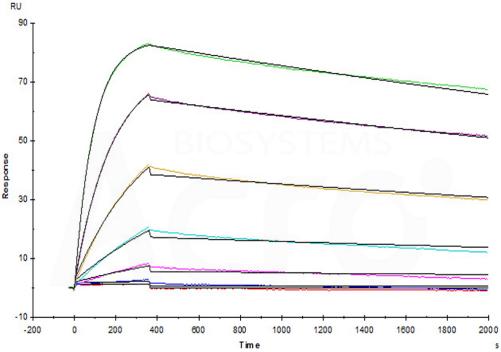


**Figure S10** The SPR analysis results published by the raw material manufacturer. The SPR affinity test results of Adalimumab and TNF-α published on the official website of the raw material manufacturer.

To facilitate a more direct comparison of the differences between MetaSPR chips and traditional SPR chips, we conducted affinity assays for the interaction between TNF-α protein and Adalimumab antibody using the CM5 chip on a Biacore T200 system. The results were then compared with those obtained from the carboxylated chip of the FED-MSPR biosensor. By comparing the real-time binding curves and simulated kinetic curves of both chips, it was observed that the response trends of the binding curves were consistent (Figure S11a, b). Upon comparing the Ka and Kd , it was found that both chips exhibited Ka and Kd values within the same order of magnitude (Table S2). A comparison of the KD values, derived from the affinity results of both chips, revealed that they were nearly identical. This finding underscores the consistency and reliability of the MetaSPR technology when compared to the gold-standard SPR technique.


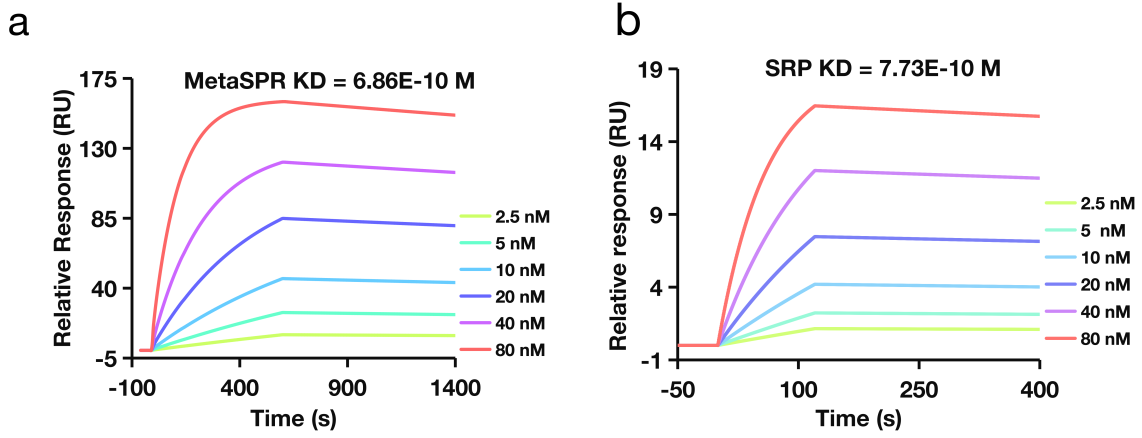


**Figure S11** Comparative Analysis of Affinity Detection Using Two Chip Types. (a) Real-time binding and simulated kinetic curves for the interaction between TNF-α protein and Adalimumab, as measured by the FED-MSPR biosensor. (b) Corresponding curves obtained using the conventional CM5 chip-based SPR sensor.

Table S2 Comparison of affinity detection results between two types of biosensors.

|  | Ka (M⁻¹s⁻¹) | Kd (s⁻¹) | KD (M) | R² |
| --- | --- | --- | --- | --- |
| FED-MSPR Biosensor | 6.70E+05 | 4.60E-04 | 6.86E-10 | 0.9956 |
| CM5 SPR Biosensor | 2.07E+05 | 1.60E-04 | 7.73E-10 | 0.9971 |

**10 Fabrication and Characterization of the FED-MSPR Biosensor**

In previously documented chip configurations, a typical Au layer thickness of approximately 70 nm or an Ag layer with a protective Au layer of approximately 20 nm thickness has been reported. The thickness of the buffer layer was set to 9 nm to provide sufficient adhesion while minimizing alterations to the fundamental SPR propagation mode through numerical simulations. Therefore, the comparison of several chips was carried out under the condition that the connecting layer was 9 nm and the total thickness of several heavy metals was 70 nm. Maintaining a consistent total chip thickness allowed for a direct comparison of the effects of the different metal layers on the SPR sensing performance. The process of making the chip is illustrated in Figure S12.


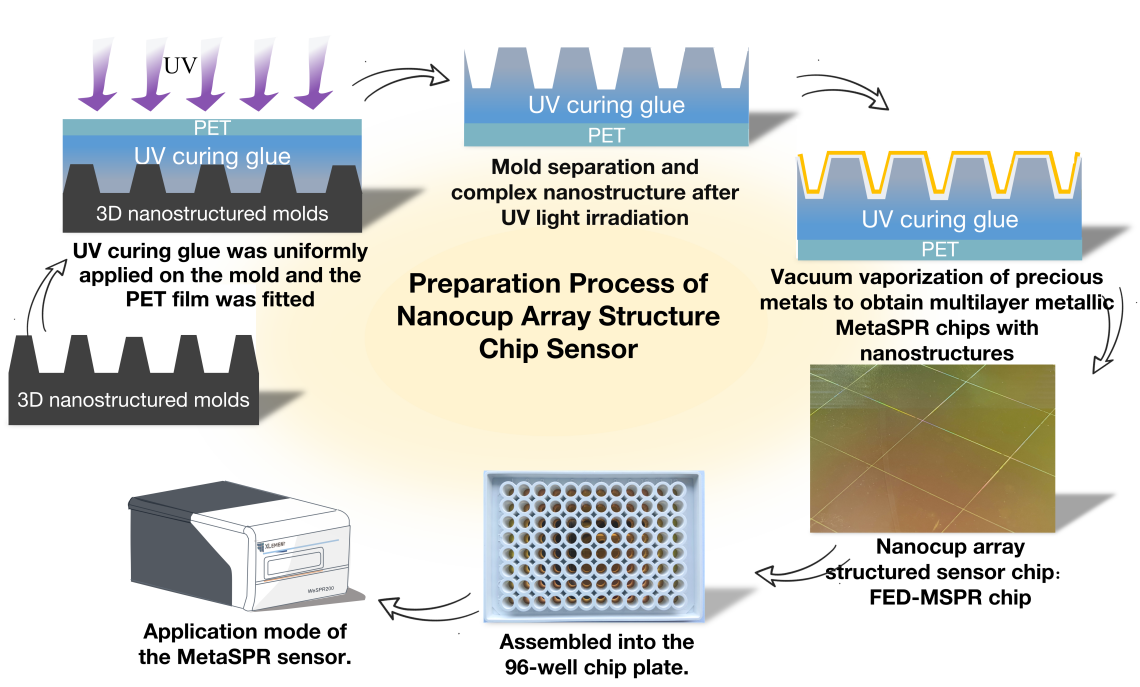


**Figure S12** Schematic diagram of the fabrication process of MetaSPR chip sensors with nanocup array structure.

**References**

[1] L. Huang, Y. Li, C. Luo, Y. Chen, N. Touil, H. E. Annaz, S. Zeng, T. Dang, J. Liang, W. Hu, H. Xu, J. Tu, L. Wang, Y. Shen, G. L. Liu, *Biosens Bioelectron* **2022**, 199, 113868.

[2] A. Ameen, L. P. Hackett, S. Seo, F. K. Dar, M. R. Gartia, L. L. Goddard, G. L. Liu, *Advanced Optical Materials* **2017**, 5, 1601051.
